# Pre-service ultrasonic and manual evaluation of the reproductive organs of dairy cows presumed to be in estrus

**DOI:** 10.1186/2193-1801-3-529

**Published:** 2014-09-15

**Authors:** Endris Hansar, Alemayehu Lemma, Tefera Yilma

**Affiliations:** College of Veterinary Medicine and Agriculture, Addis Ababa University, P. O. Box 34, Debre Zeit, Ethiopia

**Keywords:** Artificial insemination, Estrus sign, Reproductive organs, Ultrasonography

## Abstract

Manual and ultrasonic evaluation of the the reproductive organs of 62 dairy cows and heifers belonging to smallholder farms was conducted. The objective was to assess the efficiency of visual estrus detection method in the dairy animals that were presented for artificial insemination. Data were collected on reproductive status, body condition, estrus signs, and findings of rectal examination. Thirty-three animals were inseminated after ultrasonic evaluation while 29 animals were inseminated after manual evaluation through rectal palpation. Pregnancy diagnosis was performed using ultrasound 30 days post insemination. The most frequently observed estrus signs by the dairy owners were mucus discharge (83.9%) and mounting (67.7%) while the most frequently confirmed physical signs of estrus by rectal examination were cervical opening (88.7%), and uterine turgidity (82.3%). The overall mean (±SD) diameter of the largest follicle was 12.9 ± 3.4 mm with an average number of 3.5 follicles per ovary. The overall pregnancy rate was 29%. Evaluation of post-insemination records showed a significant difference (P < 0.05) in the diameter of largest follicle between the pregnant (14.7 mm) and non-pregnant (12.1 mm) animals. The mean (±SD) time interval between the first observed estrus sign to service were 10.5 ± 7.0 hrs and 14.5 ± 10.0 hrs for pregnant and non-pregnant, respectively. The low pregnancy rate, delayed time of insemination, and the difference in the size of dominant follicle indicate the incompatibility between visual estrus detection and optimal time of insemination. This confirms the significance of improving estrus detection and the need for combining estrus signs with pre-service evaluation of the reproductive organs either manually or using ultrasound.

## Introduction

Dairy productivity is a function of both lactation and reproductive performance; hence, the productive performance of a given breed of dairy cow should never be separated from the reproductive efficiency of the animal (Mukassa-Mugerwa et al. 1989) and (Negussie et al. 2002). Reproductive efficiency is known to be the product of successful estrus detection and conception rates (Cavestany and Galina 2001). Normal estrous cycles in cows and overt signs of estrus are essential, so that insemination can occur at the appropriate time (6–12 hr after onset of estrus) relative to ovulation (Dransfield et al. 1998). Estrus detection, therefore, becomes a vital aspect of dairy herd reproductive management. Whilst good estrus detection does not necessarily guarantee good reproductive performance, poor estrus detection makes poor performance hard to avoid (Arthur 2001).

Dobson et al. (2000) report that the percentage of animals that stand to be mounted has declined from 80% to 50% and the duration of detected estrus has reduced from 15 h to 5 h over the past 50 years. Poor estrus manifestation and failure to detect estrus further hinders insemination at the correct time, which is an important cause of fertilization failure (Dransfield et al*.*^1998^; Hall and Dorsey 2005). Insemination very early in estrus also causes reduced fertility, possibly due to reduced sperm survival rates before fertilization (Andrew et al. 2004). Thus, synchronizing the time of ovulation with insemination is an important factor in successful fertilization (Nebel et al. 1994) and (Dransfield et al*.*^1998^). In Ethiopia, efforts have been made to improve the productivity of indigenous zebu breeds through cross-breeding using artificial insemination (AI) to incorporate imported genetic material (Tegegne et al. 1995). However, the success of AI has not been high, and its application has been constrained by a number of factors. The use of AI as the main method of breeding dairy cows means that the responsibility for estrus detection falls upon the dairy owners who manage the herd. Among the technical constraints are poor heat detection skills, poor semen quality, inefficiency of AI technicians, and communication and transport problems (Belihu 2002), and (Gebremedhin 2008). The objectives of the present study were, therefore, to evaluate the efficiency of estrus detection by dairy owners in the success of AI and identify the role of manual and ultrasonic evaluation of reproductive organs before AI in improving pregnancy rate.

## Materials and methods

### Study area and animals

The study was conducted at the veterinary clinic of the school of veterinary medicine in the town of Debre Zeit, located 45 km southeast of the capital Addis Ababa. It has an altitude of 1990 meters, annual rainfall of 866 mm, mean relative humidity of 61.3%, and annual minimum and maximum temperatures of 14°C and 26°C, respectively.

Sixty-two crossbred dairy cattle (26 heifers and 36 cows) that belonged to private smallholder dairy farms and presented for AI at the veterinary clinic were included in the present study. All the cows and heifers were transported to the clinic for insemination. Decision as to whether to pursue a manual or ultrasonic examination before the insemination was made randomly after the animals were presented at the clinic. Accordingly, 33 animals (18 cows and 15 heifers) were randomly selected for ultrasonic examination while the remaining 29 animals were only manually examined. All the animals were properly housed, supplemented with hay and concentrate, and had free access to water on daily basis. The average body condition score (BCS) was determined on a 0 to 5 scale (Buckley et al. 2003). All the farms practiced deworming and vaccination for common infectious diseases. AI service was performed upon the owner’s request after a visual detection of presumed estrus signs.

### Study design and data analysis

#### Records of reproductive status

Information about the age of the animals, parity, estrus signs manifested before the animal was brought for insemination, data on duration of estrus signs, number of previous inseminations, and milk yield were collected. Each animal was then physically examined for general health before insemination. Owners were additionally interviewed about their knowledge of estrus signs and their breeding practices.

#### Evaluation of the reproductive organs

The manual examination involved checking for pregnancy, for the symmetry, and turgidity of the uterine horns or for the presence of any pathological conditions. The ovaries were also palpated for presence of mature follicles, cysts, or corpus luteum (CL). The animals selected for the ultrasonography (n = 33) were evaluated using ultrasound scanner with a 5 MHz linear array transducer (Aloka, Japan). The echotexture of the uterine wall, presence of fluid or any secretion, and presence of pregnancy were assessed. Both left and right ovaries were examined for presence of follicles or CL. The number of follicles were counted, and diameters of the three largest follicles in each ovary were measured using the internal electronic caliper. Ovarian follicular data were later compared to pregnancy rate.

#### AI and pregnancy diagnosis

All the animals that were confirmed by the owner to have shown estrus signs and that did not present any apparent pathological condition during rectal or ultrasonic examination were approved for insemination. Semen procured from the National Artificial Insemination Center (NAIC) was thawed for 30 sec at 35°C before insemination. The perineum was cleaned and towel-dried before semen was deposited close to the internal *os* of the cervix about 1 cm into the body of the uterus as in (Dransfield et al*.*^1998^). Pregnancy was diagnosed by ultrasound on Day 30 post-insemination.

All data were entered to Microsoft Excel (2003) for analysis with SPSS for Windows (Version 15, USA). Data were summarized using descriptive statistics. Proportions were compared using Chi-square test. The relationships between variables were computed using Pearson correlation. Effects of time from estrus to AI and size of the largest follicles on pregnancy rate were computed using student *t*-test and ANOVA. The level of significance was held at p < 0.05 to show statistically significant differences among variables.

## Results

### Estrus signs and time of insemination

The most frequent and consistent estrus signs reported by the dairy owners were mucus vaginal discharge, mounting other animals and bellowing (Table 1). The mean (±SD) time interval between observation of estrus signs and insemination (n = 62) was 13.3 ± 9.3 hrs. None of the owners reported routinely practicing scheduled estrus detection or pregnancy diagnosis. However, owners do make visual assessments during the afternoon milking. Decisions as to when to take the animals for insemination varied among the owners. Some owners often wait until the estrus signs they believe to be best appear; whereas, a few others do not believe time of insemination to be important, so they bring their animals when convenient.

**Table 1:** **Most common estrus signs observed by dairy owners and approximate time to insemination**

Estrus sign	Overall (n = 62)	Manually examined (n = 29)	Ultrasound examined (n = 33)	Pregnant (n = 18)	Non-pregnant (n = 44)
Bellowing [%]	60.0	51.7	54.5	61.1	59.5
Mounting others [%]	66.7	48.3	69.7	77.8	64.2
Mucus vaginal discharge [%]	83.3	79.3	87.9	88.9	83.3
Estrus to AI [hrs]	13.3 ± 9.3	13.4 ± 9.7	12.1 ± 9.6	8.5	15.10

Out of the 33 ultrasonically evaluated animals, 20 (60.6%) showed both mucus vaginal discharge and mounting others. Mean (±SD) diameter of the largest follicle for animals showing both signs was 12.7 ± 4.4 mm. Dairy owners believed vaginal mucus discharge and mounting other animals to be the best signs of estrus. Nine, five and six animals showing these two signs were brought for insemination within 6 hrs, 7–14 hrs and after 14 hrs of observed heat, respectively, with mean follicle sizes of 13.5 ± 3.2 mm, 13.0 ± 4.7 mm, and 11.1 ± 5.8 mm, respectively.

The average time to AI for animals showing both mucus discharge and mounting that later became pregnant was 6.4 hrs with 13.8 mm average diameter of the dominant follicle. In contrast, non-pregnant animals showing both mucus and mounting were brought for AI after 14.1 hrs, with average diameter of the largest follicle at 9.7 mm. The average body condition score (BCS) for all study animals was 3.0 ± 0.51. BCS was not significantly different between pregnant (3.0 ± 0.51) and non-pregnant (2.6 ± 0.50) animals.

### Pregnancy rate in rectally evaluated animals

The most frequent and consistent findings of the rectal examination (n = 62) were open cervix and uterine turgidity. The total number of pregnant animals was 18 (30%) and non-pregnant 44 (70%). Although only 39.4% (13/33) of the animals were found to be at the right stage for insemination during ultrasound evaluation, all were inseminated. Among the 25 non-pregnant animals evaluated by ultrasound, 64% showed mounting. The average time from detection of estrus signs to insemination for non-pregnant animals was 15.1 hr, and average diameter of largest follicles at the time of insemination was 10.3 mm. Eighty-four percent (21/25) of the non-pregnant animals showed mucus vaginal discharge. These animals, brought for insemination within 9.8 hrs, had average follicle size of 12.4 mm. Overall, 88.9% of pregnant animals (n = 18) showed both mounting and mucus vaginal discharge. The proportion of cows and heifers evaluated for different physical signs of estrus during rectal examination are summarized in Table 2.

**Table 2:** **Physical signs of estrus in pregnant and non pregnant animals**

Types of physical sign of estrus	Overall [%] (n = 62)	Pregnant [%] (n = 18)	Non-pregnant [%] (n = 44)
Uterine turgidity	82.3	85	81
Palpable ovarian follicles	67.7	73.8	56
Open cervix	88.7	93.75	90

### Follicular activity relative to time of insemination

Over half (56.3%) of the follicles measured were 10–15 mm in diameter with remaining 15.6% and 28.1% of the follicles being less than 10 mm and greater than 15 mm in size, respectively. The mean (±SD) diameter of the largest follicle was 12.9 ± 3.4 mm, with an average of 3.5 follicles per ovary (n = 33). There was a significant difference between heifers and cows in the mean diameter of the largest follicle (11.4 ± 3.5 mm and 14.0 ± 2.8 mm, respectively).

**Figure 1:**
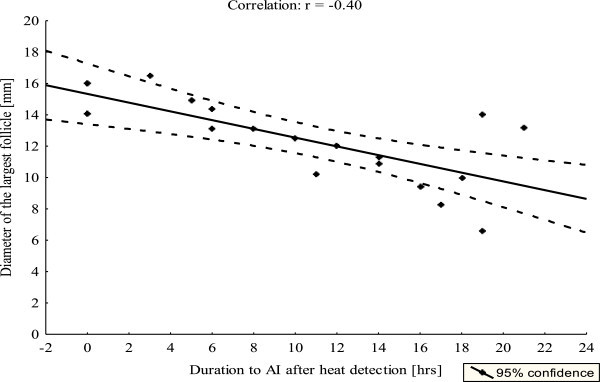
* **Correlation between the diameter of the largest follicles and the duration to AI after estrus was detected.** *(* *Plot represents 27 animals and was constructed after removing outlier data points).

Category of follicular diameter based on the time to insemination is presented in Table 3. Uterine turgidity and diameter of largest follicle were correlated (r = 0.28, p < 0.05), as was open cervix and diameter of largest follicle (r = 0.59, p < 0.001). Among behavioral signs of estrus, mounting and diameter of largest follicle were correlated (r = 0.40, p < 0.05). However, diameter of largest follicle and time to AI were negatively correlated (Figure 1).

**Table 3:** **The mean diameter of the dominant follicle at different time interval of insemination**

Duration to AI after heat detection	N	Mean (±SD) diameter of dominant follicle [mm]	Level of significance
Overall	33	12.7 ± 4.0	P < 0.05
≤6 hrs	12	12.8 ± 3.2	
7-14 hrs	7	14.7 ± 1.8	
≥15 hrs	13	10.5 ± 5.0

Similarly, diameter of the largest follicle was correlated (r = −0.31, p < 0.05) with time to insemination for pregnant animals showing all the physical signs of estrus. There was a significant (p < 0.05) difference in the diameter of the largest follicle between animals that showed mounting and those that did not, and between those with open versus closed cervix.

The proportions of pregnant heifers and cows were 38.9% and 61.1%, respectively. Pregnancy rate among animals evaluated by ultrasound was 24%. There was a significant difference in the mean diameter of largest follicle between pregnant (14.7 ± 3.3 mm) and non-pregnant animals (12.1 ± 3.37 mm) and in the time from detection of estrus to AI between successful (10.5 ± 6.9 hrs) and failed pregnancies (14.5 ± 9.9 hrs).

## Discussion

The present study revealed that dairy owners were mostly dependent on estrus signs like bellowing, mucus vaginal discharge, and mounting. The mean diameter of the dominant follicle in this study was comparable to other studies (Santos Filho et al. 2001), (Lucy 2001) and (Evans 2003). However, larger diameters between 15 and 25 mm at ovulation have also been reported (Arthur 2001) and (Peters and Benboulaid 1998). Factors such as difference in breed, geography, and management of the animals are reported reasons for variation in follicular diameter.

Various studies (López-Gatius and Camón-Urgel 1991), (Arthur 2001), (Hunter 2003) and (Roelofs et al. 2005) confirm that a cow can be classified as ready for service when the largest follicle has an estimated diameter of 12–25 mm, with slight bulging of the follicle when palpated, CL less than 10 mm or non-detectable, uterus highly turgid and contractile to the touch, and copious to transparent vaginal discharge. Considering both the pregnant and non-pregnant animals in this study, the majority were presented by their owners either too early or too late relative to the time of ovulation. Although clear mucous vaginal discharge is a relatively reliable sign of estrus, particularly in continuously cows, the timing of its appearance varies, and insemination could be mistimed by up to two days (Arthur 2001). In this study, mounting other animals was observed relatively late compared to vaginal mucous discharge.

Bull sperm cells take several hours in the cow’s genital tract before they are capable of fertilizing the ovum (Andrew et al. 2004). Ovulation occurs about 10 hr after standing estrus in normal cows, and the unfertilized eggs can survive in the cow’s oviduct for only few hours (Roelofs et al. 2005). Therefore, inseminating too long before ovulation can result in lower conception rates, because the ovum can be dead before the sperm are capable of fertilization.

The pregnancy rate obtained in this study was comparable with low rates reported in both Ethiopia (Gebremedhin 2008); (Lemma and Kebede 2011) and elsewhere (Lucy 2001). The result was, however, much lower than the 41 to 48% reported by (Shiferaw et al*.*^2003^) and (Lobago et al. 2006). Previous studies on the effect of mating system and farm size showed that smaller farms using AI have higher service per conception (2.9 times/conception) and lower pregnancy rate (20%) compared to farms using natural service (1.7 and 40%, respectively) that can rely on estrus detection by the bull (Lemma and Kebede 2011). Manual palpation (López-Gatius and Camón-Urgel 1991) or transrectal ultrasonography of the bovine reproductive tract allows more accurate diagnosis of estrus when the animal is ready for service (Roelofs et al. 2004).

## Conclusion

Poor timing between ovulation and AI seems to be the main factor that influenced the pregnancy rate in this study. AI either too early or too late was associated with the absence of one or more of the behavioral and physical signs, with smaller, developing follicles or follicles that had already ovulated. It is clear that lack of awareness by the dairy owner seriously affects efficiency of AI service in smallholder dairy production. Hence, it is important to train dairy owners how to detect estrus by scheduled regular observations to improve its timely detection. Furthermore, pre-service evaluation of the female reproductive tract, by ultrasound, manual palpation or both, can reduce service failure. Ultrasound equipment is rather expensive for routine application, but manual palpation could be combined with improved estrus detection to improve the success of AI and increase pregnancy rates.

## Authors’ information

EH DVM, instructor in veterinary general surgery; AL PhD, Associate professor in veterinary obstetrics and gynecology; TY PhD and Assistant professor in veterinary obstetrics and gynecology.

## Abbreviations

AI: Artificial insemination
BCS: Body condition score
NAIC: National Artificial Insemination Center.
